# Neural Mechanisms of Social Interaction Perception: Observing Interpersonal Synchrony Modulates Action Observation Network Activation and Is Spared in Autism

**DOI:** 10.1002/hbm.70052

**Published:** 2024-10-24

**Authors:** Afton M. Bierlich, Nanja T. Scheel, Leora S. Traiger, Daniel Keeser, Ralf Tepest, Alexandra L. Georgescu, Jana C. Koehler, Irene Sophia Plank, Christine M. Falter‐Wagner

**Affiliations:** ^1^ Department of Psychiatry and Psychotherapy LMU University Hospital, LMU Munich Munich Germany; ^2^ NeuroImaging Core Unit Munich (NICUM) LMU University Hospital, LMU Munich Munich Germany; ^3^ Department of Psychiatry and Psychotherapy, Faculty of Medicine and University Hospital Cologne University of Cologne Cologne Germany; ^4^ Thymia Limited London UK; ^5^ Department of Psychology Institute of Psychiatry, Psychology and Neuroscience, King's College London London UK

**Keywords:** Action Observation Network, autism, fMRI, interpersonal synchrony perception, social interaction perception

## Abstract

How the temporal dynamics of social interactions are perceived arguably plays an important role in how one engages in social interactions and how difficulties in establishing smooth social interactions may occur. One aspect of temporal dynamics in social interactions is the mutual coordination of individuals' behaviors during social interaction, otherwise known as behavioral interpersonal synchrony (IPS). Behavioral IPS has been studied increasingly in various contexts, such as a feature of the social interaction difficulties inherent to autism. To fully understand the temporal dynamics of social interactions, or reductions thereof in autism, the neural basis of IPS perception needs to be established. Thus, the current study's aim was twofold: to establish the basic neuro‐perceptual processing of IPS in social interactions for typical observers and to test whether it might differ for autistic individuals. In a task‐based fMRI paradigm, participants viewed short, silent video vignettes of humans during social interactions featuring a variation of behavioral IPS. The results show that observing behavioral IPS modulates the Action Observation Network (AON). Interestingly, autistic participants showed similar neural activation patterns as non‐autistic participants which were modulated by the behavioral IPS they observed in the videos, suggesting that the perception of temporal dynamics of social interactions is spared and may not underly reduced behavioral IPS often observed in autism. Nevertheless, a general difference in processing social interactions was found in autistic observers, characterized by decreased neural activation in the right middle frontal gyrus, angular gyrus, and superior temporal areas. These findings demonstrate that although the autistic and non‐autistic groups indeed differed in the neural processing of social interaction perception, the temporal dynamics of these social interactions were not the reason for these differences in social interaction perception in autism. Hence, spared recruitment of the AON for processing temporal dynamics of social interactions in autism does not account for the widely reported attenuation of IPS in autism and for the widely reported and presently observed differences in social interaction perception in autism.

## Graphical abstract

Using task‐based fMRI, we investigated the neural basis of interpersonal synchrony (IPS) perception. Our findings show that IPS modulates neural activation in the Action Observation Network when individuals observed human social interactions. These activation patterns were comparable for autistic and non‐autistic individuals, suggesting that IPS perception is spared in autism.

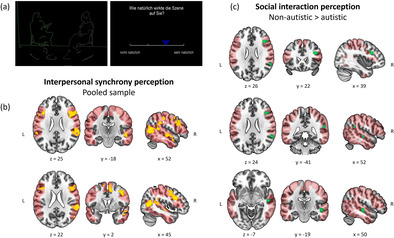

## 1 Introduction

Observing others in social interactions largely informs how one learns to interact with others (Bandura 1977). Recently, increasing research interest has led to studies beginning to unravel how humans neurally process social interactions they observe; arguably, a fundamental aspect of one's ability to engage in social interactions (Abassi and Papeo 2022; Centelles et al. 2011; Isik et al. 2017; Landsiedel et al. 2022; Quadflieg and Koldewyn 2017; Walbrin, Downing, and Koldewyn 2018; Walbrin and Koldewyn 2019). As crucial components of social interaction perception, extensive neuroimaging research has mapped the neural representations of action observation and execution (for a review, see Hardwick et al. 2018), subsequently defining a collection of regions known as the Action Observation Network (AON). The AON includes the bilateral posterior superior temporal sulcus (pSTS), inferior parietal lobe (IPL; including the supramarginal and angular gyri), inferior frontal gyrus (IFG), and pre‐motor areas including the precentral and postcentral gyri as well as the supplementary motor area (SMA) (Georgescu et al. 2014). Activation of AON‐associated regions, specifically the lateral occipital and superior temporal regions, has been shown for the observation of dyadic interactions compared with two agents acting independently (Isik et al. 2017; Walbrin and Koldewyn 2019) or non‐facing dyads (Abassi and Papeo 2022). As a relatively new avenue of investigation in general, little research has directly investigated how the neural basis of dynamic social interaction perception may differ in disorders characterized by differences in social behavior, such as autism. Drawing from the broader social domain, some evidence points to altered AON activation for social cognition and behavioral imitation in autism (Kilroy, Cermak, and Aziz‐Zadeh 2019; although, see Pokorny et al. 2018). It is therefore plausible that potential differences in social interaction perception by autistic individuals could be reflected in the neural activation of the AON. Moreover, the AON's involvement in social interaction perception indicates that it could be a candidate network engaged in timing perception when observing others' behaviors, namely behavioral interpersonal synchrony (IPS), an aspect of behavior in interactions known to be reduced in autism (McNaughton and Redcay 2020).

Behavioral IPS is the temporal coordination of individuals' behaviors during social exchange, which has been shown to facilitate natural social interactions (Bowsher‐Murray et al. 2022; Hoehl, Fairhurst, and Schirmer 2021; Hu et al. 2022) and strengthen social bonds (Hove and Risen 2009; Marsh, Richardson, and Schmidt 2009; Miles, Nind, and Macrae 2009). Nonverbal behaviors, such as gestures and other body movements, tend to synchronize with or mimic those of an interaction partner, which contributes to establishing smooth interactions (Chartrand and Bargh 1999; Vicaria and Dickens 2016). Considering these prosocial implications, it is essential to investigate the neural basis of IPS in social interaction perception to understand how humans establish temporal coordination of behavior when they interact with others and how difficulties in establishing synchrony may occur.

Few studies have investigated the neural basis of social interaction perception with a focus on temporal features observed in others' behaviors. Georgescu et al. (2014) investigated how dyadic movement velocity and contingency of animated virtual characters are perceived and found increased activation within the AON, specifically the bilateral pSTS, IFG, IPL, and occipital–temporal cortex, when observing contingent (i.e., temporally coordinated), compared to mirrored, interactions. More recently, Tsantani, Yon, and Cook (2024) found activation of the STS, extrastriate body area (EBA; including part of the lateral occipital cortex [LOC]), and the fusiform face area (FFA) when individuals observed interacting animated virtual characters' synchronous compared to asynchronous head movements. However, it is not yet known how findings regarding perceiving virtual character interactions map onto the perception of human–human interactions in general and specifically with respect to IPS. Thus, the present study examined the perception of temporal dynamics in social interactions from a neuroimaging perspective, specifically investigating the neural mechanisms underlying IPS in social interactions when one is observing social interactions between humans with varying behavioral IPS.

Given the inherent social interaction difficulties in autism, IPS has recently been investigated by an increasing number of studies, which show reduced IPS during social interactions including an autistic individual (McNaughton and Redcay 2020), for instance, with respect to movement synchrony (Carnevali et al. 2024). Dyads including an autistic individual tend to produce less movement IPS than dyads of two non‐autistic individuals in both children (Su, Culotta, Hoffman, et al. 2020; Su, Culotta, Mueller, et al. 2020; Zampella et al. 2020) and adults (Georgescu et al. 2020)—a pattern that appears to be specific to autism compared with differential diagnoses also associated with social interaction difficulties (Koehler et al. 2021). Notably, some evidence demonstrates a preference for interacting with someone with the same diagnostic status (Crompton, Ropar, et al. 2020; Crompton, Sharp, et al. 2020; Morrison et al. 2020). However, it has been previously shown that the variability of intra‐individual timing during communication is increased in autistic individuals (Bloch et al. 2022), which makes behavioral alignment in homogeneous autism dyads unlikely. Evidence has further shown that increased variability in intra‐individual timing during communication leads to increased decoding costs for the observing interaction partner (Bloch et al. 2024). These decoding costs were higher for autistic individuals when observing the autistic communication style, suggesting a potential disadvantage when an autistic person decodes an autistic communication style (Bloch et al. 2024). Although social interactions between individuals sharing diagnostic status might be preferred (Morrison et al. 2020), this preference is not reflected in a shared‐diagnosis communication advantage (Bloch et al. 2023, 2024). Moreover, behavioral IPS was reduced for dyads including autistic individuals, regardless of the interaction partner's diagnostic status (Georgescu et al. 2020). Indeed, difficulties with reciprocity in social interactions are a defining symptom of autism, and hence an interaction between two individuals who both have difficulties with reciprocity would be arguably unlikely to lead to higher IPS.

Importantly, the underlying mechanisms of such behavioral IPS attenuation have not yet been revealed. One possible root of this attenuation could indeed stem from differentiated *perception* of the timing of others' behaviors, as synchronization is inherently rooted in temporal processing.

Differences in timing functions (e.g., duration discrimination, simultaneity detection, motoric timing), including partially superior performance patterns, have been shown to be part of the autistic cognitive profile (Allman 2011; Allman and Falter 2015; Bebko et al. 2006; Boucher et al. 2007; Brodeur et al. 2014; Falter, Elliott, and Bailey 2012; Falter et al. 2013; Falter, Noreika, et al. 2012; Falter and Noreika 2014; Gepner and Feron 2009; Gowen and Miall 2005; Isaksson et al. 2018; Karaminis et al. 2016; Kargas et al. 2015; Kwakye et al. 2011; Maister and Plaisted‐Grant 2011; Martin, Poirier, and Bowler 2010; Stevenson et al. 2016; Szelag, Kowalska, and Galkowski 2004; Welsh, Ahn, and Placantonakis 2005; Wimpory, Nicholas, and Nash 2002). However, differences in other timing functions (e.g., relative timing, duration perception, clock processes) in autism were not unequivocally found (e.g., Bebko et al. 2006; Gil et al. 2012; Glazebrook, Elliott, and Lyons 2008; Isaksson et al. 2018; Jones et al. 2009; Jones, Lambrechts, and Gaigg 2017; Kwakye et al. 2011; Mostofsky et al. 2000; Poole et al. 2022; Wallace and Happé 2008). If the timing of others' behaviors is processed differently by autistic individuals at the neural level, this could be associated with attenuated IPS execution. Clarifying whether the neural processing of IPS in social interaction perception functions differently in autism would shed light on the role of perception as a potential explanation for attenuated behavioral IPS and, in a wider consequence, for social interaction difficulties.

Thus, the aim of the present preregistered study was twofold: (i) to establish the neural correlates of IPS in social interaction perception and (ii) to investigate whether such neural mechanisms might differ in autistic, as compared with non‐autistic, individuals to offer a potential explanation for attenuated behavioral IPS. We hypothesized that neural activation of the AON would be modulated by varying behavioral IPS that was observed in vignettes of human dyadic social interactions and that activation patterns would be differentiated for autistic compared to non‐autistic individuals during perception of social interactions that vary in terms of IPS.

## 2 Methods

### 2.1 Participants

The present study was part of a larger experimental session (preregistration: osf.io/u2j8m) and was approved by the ethics committee of the Medical Faculty at LMU (No. 20‐1050). Participants provided informed consent, in accordance with the Declaration of Helsinki (World Medical Association 2013), and were monetarily compensated for their participation.

We recruited 62 individuals (33 autistic, 29 non‐autistic) via in‐house, local, and regional channels. Autistic participants had a confirmed ICD‐10 diagnosis of F84.5, F84.0, or F84.1 (World Health Organization 2016). Non‐autistic participants had no psychiatric diagnoses as per self‐report. Inclusion criteria were ages between 18 and 60 years old, no neurological diagnoses, no metal or cochlear implants as well as pacemakers, an IQ above 70, and normal or corrected‐to‐normal vision. The Mehrfachwahl‐Wortschatz‐Intelligenztest (MWT‐B; Lehrl et al. 1999) assessed verbal IQ, and the Culture Fair Test (CFT‐20‐R; Weiß 2006) assessed nonverbal IQ.

Data from three non‐autistic individuals were omitted after piloting, and data from one autistic individual was excluded due to an IQ below 70. Data from seven individuals (one non‐autistic, six autistic) were excluded due to excessive head movement while in the scanner. The analyzed sample consisted of data from 26 autistic (9 identified as female, 17 as male) and 25 non‐autistic individuals (8 identified as female, 17 as male). Groups were matched for age and IQ but expectedly differed in social traits and motor coordination (Table 1), as measured by the German translations of the Autism Spectrum Quotient (AQ; Baron‐Cohen et al. 2001) and Adult Dyspraxia Checklist (ADC; Kirby et al. 2010), respectively.

**TABLE 1:** Means and standard deviations are reported for each group, as well as the log‐transformed Bayes factor (Log(BF_*10*_)) from a Bayesian Mann–Whitney U test (^a^) or Bayesian independent samples *t*‐test (^b^).

	Autistic	Non‐autistic	Log(BF_*10*_)
Age^a^	35 ± 11.46	34.8 ± 10.65	−1.240
CFT‐20‐R^b^	111 ± 21.06	120.08 ± 18.81	−0.202
MWT‐B^a^	110.96 ± 12.20	117.4 ± 15.02	−0.259
AQ^a^	35.58 ± 6.75	16.04 ± 6.75	7.298
ADC^b^	58.92 ± 17.73	18 ± 8.71	25.115

### 2.2 Experimental Design and Procedure

**FIGURE 1:**
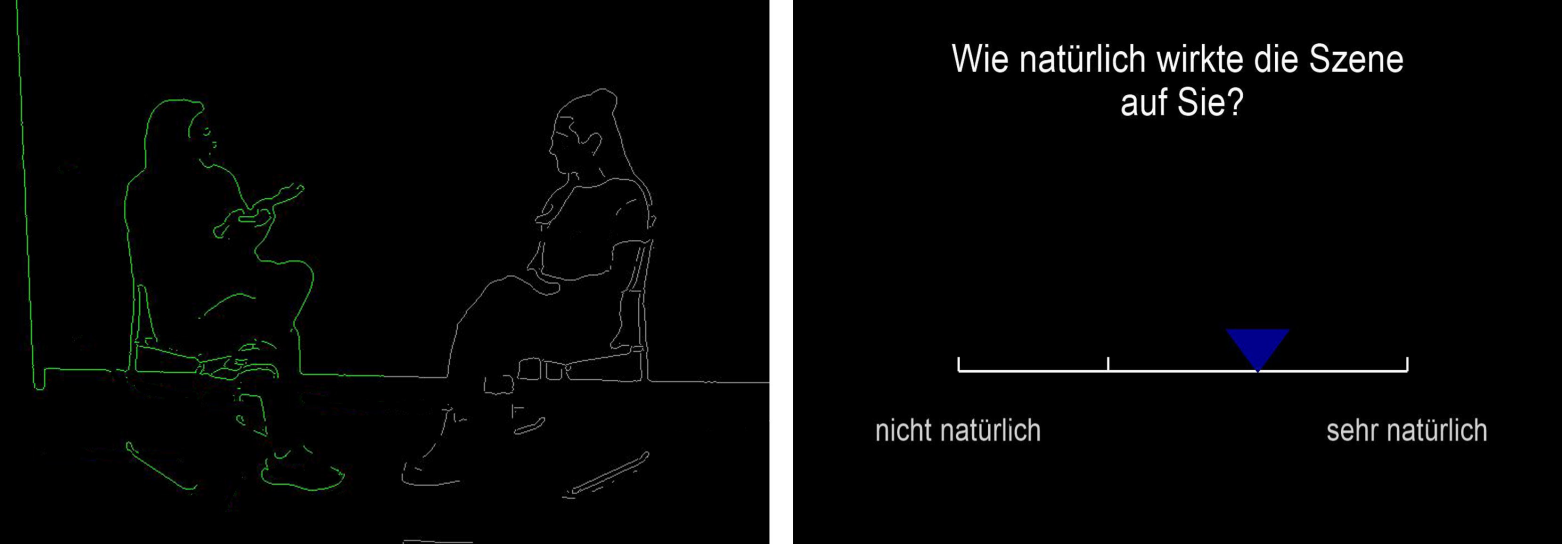
A screenshot from an example video vignette (left) which is followed by the rating question, “How natural did you find the scene?” and the response options between “not natural” and “very natural” (right). The video vignettes varied in the amount of behavioral IPS that the depicted dyad produced. Participants were not made aware that the dyads they observed varied in behavioral IPS. The rating question served as a check to ensure that participants actively paid attention to the task and as an assessment of the social scene. This image has been recreated with lab personnel to closely resemble the video vignettes used in the task.

We adapted the experimental design employed by Georgescu et al. (2014). Participants viewed 44 silent video vignettes of interacting dyads with varying behavioral IPS on a continuum as extracted from the video vignettes. The extracted IPS served as the independent variable (within‐group) in the present study. Each video was followed by a rating scale asking how natural participants found the scene (Figure 1) using a 4‐point Likert scale (1 = *not natural*; 4 = *very natural*), whereby the starting position of the marker was random. This behavioral rating was used to ensure that participants were attending to and actively watching the videos, serving as an assessment of the social scene. We opted for a general rating of the social scene, rather than IPS, because we aimed to assess how behavioral IPS *implicitly* modulates perception at the neural level. Each trial included a jittered interstimulus interval (ISIs: 1.5, 1.75, 2.25, 2.5 s) between the video and rating scale and a jittered intertrial interval (ITIs: 5.4, 6.3, 8.1, 9.0 s) between the trials.

Participants completed a training session to familiarize themselves with the response boxes before entering the scanner. In the scanner, participants performed the observational task while their neural activity was recorded.

As this task was part of a larger experimental protocol, it was counterbalanced with another fMRI task (Bierlich, Plank, et al. 2024). In total, the scanning session for both tasks lasted approximately 40 min. Participants also completed behavioral tasks and various questionnaires that will be reported elsewhere.

Of relevance for the present study, one of the behavioral tasks was a naturalistic conversational task where participants interacted with a confederate, who was naïve to the participant's diagnosis. The interactions were video recorded, which allowed for later computation of behavioral IPS for the participant and their interaction partner in the same way behavioral IPS was extracted from the videos in the present study. Notably, behavioral IPS produced by autistic participants and their interaction partners was reduced compared with non‐autistic participants and their interaction partners (Bierlich, Scheel, et al. 2024). For further details concerning the behavioral study, see Bierlich, Scheel, et al. (2024).

### 2.3 Behavioral IPS of Stimuli

The present stimulus set included 44 videos with varying amounts of behavioral IPS (Supporting Information S1). Each silent video vignette was 10 s long and depicted two individuals having a conversation (Figure 1). Participants did not receive any information on the individuals, context, or content of the conversations depicted. Participants were also not made aware that the dyads they observed varied in behavioral IPS. The dyads included either two non‐autistic individuals (non‐autistic dyad) or an autistic and a non‐autistic individual (mixed dyad). The video excerpts were selected from previous studies measuring behavioral IPS in a conversation task (Georgescu et al. 2020; *n* = 24 excerpts) and in a clinical conversation (Koehler et al. 2021; *n* = 20 excerpts). The videos were masked to only depict the outlines of each interactant's body and thereby capturing the movement and IPS of the interactants (Figure 1). Because one interactant was holding a clipboard in some vignettes (Koehler et al. 2021), we assessed the synchrony of interactants' head movements. For each vignette, motion energy was measured using Motion Energy Analysis (Ramseyer 2020), and IPS of motion energy was computed with window lagged cross‐correlations using rMEA (Kleinbub and Ramseyer 2021), resulting in a continuum of behavioral IPS values. Behavioral IPS was compared with generated pseudosynchrony to establish efficacy in the present stimulus set. Plank et al. (2023) further elaborate on the development of this stimulus set.

### 2.4 fMRI Data Acquisition

Data were collected at the NeuroImaging Core Unit Munich (NICUM) at LMU Munich using a 3 Tesla MRI scanner (Siemens Magnetom Prisma, Siemens Medical Solutions, Erlangen, Germany). Structural T1‐weighted scans were collected (176 slices; voxel size = 1 mm^3^; TR = 2250 ms; FOV = 256 mm) followed by field maps (32 slices; voxel size = 3 mm^3^; TR = 8000 ms; TE = 66 ms; FOV = 210 mm). Then, T2‐weighted echo‐planar imaging (EPI) sequences measured brain activation during the observational fMRI task (maximum 610 volumes; 32 slices; voxel size = 3 mm^3^; TR = 2066 s; TE = 30 ms; FOV = 210 mm).

### 2.5 Behavioral and fMRI Preprocessing

Creation of the fMRI time series and preprocessing of the behavioral ratings was conducted in RStudio 2023.6.0 (Posit Team 2023), using R 4.2.2 (R Core Team 2022). Twelve participants were missing one behavioral rating, and one participant was missing three behavioral ratings.

NIFTI conversion was conducted using dcm2bids 2.1.6 (Bedetti et al. 2021). fMRI pre‐processing was performed using fMRIPrep 22.1.1 (Esteban et al. 2019). The automatically generated template from fMRIPrep details the preprocessing pipeline and is reported in Supporting Information S2. The T1‐weighted anatomical scans were used as reference images after undergoing correction for non‐uniformity, skull‐stripping, brain tissue segmentation, surface reconstruction (FreeSurfer 7.2.0; Dale, Fischl, and Sereno 1999), spatial normalization, and registration to MNI152 standard space templates. Each EPI scan underwent field map correction, slice time correction, and co‐registration to the T1‐weighted reference. Spatial smoothing (Gaussian kernel 6 mm FWHM) and motion artifact removal using ICA‐AROMA (Pruim et al. 2015) were performed. Data from seven participants were omitted because participants' head motion exceeded more than one voxel (3 mm) for any of the three translational head motion parameters. The resampled brain mask was applied to the preprocessed EPI scans using fslmaths (Smith et al. 2004).

### 2.6 Behavioral and fMRI Analysis

fMRI analysis was performed using FSL FEAT (Smith et al. 2004). At the subject‐level, a general linear model was constructed using a block design including separate regressors with behavioral IPS and motion quantity extracted from the observed stimuli as continuous parametric modulators as well as a regressor with no parametric modulator. Contrasts were created to assess neural activation as modulated by behavioral IPS from the observed stimuli, indexing the perception of temporal dynamics of social interactions, and neural activation of the task, as a measure of general social interaction perception. At the group‐level, one‐sample *t*‐tests assessed the effect of observed behavioral IPS modulation in a pooled sample, and unpaired *t*‐tests compared the autistic and non‐autistic groups for the observed behavioral IPS modulation and the effect of task. Regions of interest (ROI) were defined using Marina in a single mask (Walter 2003). The mask (Supporting Information S3) covered the AON and occipital‐temporal areas as reported by Georgescu et al. (2014). This included the bilateral IFG, superior temporal gyrus (STG), IPL and supramarginal gyrus (SMG), middle temporal gyrus (MTG), inferior temporal gyrus (ITG), as well as the SMA and the precentral and postcentral gyri.

A whole brain approach also explored activation outside of the defined ROIs. A whole brain approach was used to further explore whether the neural mechanisms underlying IPS in social interaction perception when observing others in social interactions was associated with behavioral IPS produced when involved in naturalistic social interactions. We modeled the association of the neural correlates of observed IPS with the behavioral IPS produced by the same participants in the conversation task (Bierlich, Scheel, et al. 2024), both within the pooled sample and between groups. Finally, whole brain analyses explored the effect of dyad composition observed in the video vignettes, resulting in two differential contrasts at the subject‐level (mixed > non‐autistic and non‐autistic > mixed). One sample *t*‐tests assessed each of the differential contrasts in a pooled sample, and unpaired *t*‐tests compared the autistic and non‐autistic groups by stimulus dyad composition.

Group‐level analyses were conducted using a non‐parametric approach with FSL randomise (5000 permutations, *p* < 0.05). Using TFCE thresholding, cluster leakage (Spisák et al. 2019) was observed (Supporting Information S4). Remaining in line with our cluster‐based approach, we opted for cluster mass thresholding to allow inferences about significant clusters. Identified brain regions are specified from the Harvard‐Oxford Atlas and figures were created using MRIcroGL (Rorden and Brett 2000). Quality metrics (MRIQC; Esteban et al. 2017) and structural analyses (NAMNIs; Karali et al. 2021) are reported in Supporting Information S5 and S6.

A Bayesian linear mixed model with a cumulative probit distribution was conducted to evaluate the behavioral ratings using the brms package (Bürkner 2017). The model included diagnostic group (autistic/non‐autistic) and centered behavioral IPS extracted from the video vignettes as predictors of interest, motion quantity extracted from the video vignettes as a predictor of no interest, and participant as a random intercept. Sum‐contrast coding was used for diagnostic group. As preregistered, we expected that behavioral IPS as observed in the video vignettes might differentially influence ratings in autistic individuals compared to non‐autistic individuals.

Computational faithfulness and model sensitivity were evaluated using the Simulation‐based Calibration package (SBC; version 0.2.0.9000; Modrák et al. 2023). Weakly informative priors were set individually for each intercept, prior (−0.67, 1), prior (0, 1), prior (0.67, 1), to model the probability of each rating category. Weakly informative priors were also set for beta parameters, prior (0, 1), and the random intercept, prior (0, 1). The model was run over 300 simulated data sets with four Markov chains, 6000 iterations (25% warm‐up), and an initial value set to 0.1 for all parameters. Posterior ranks were visually inspected before running the same model on the true data. Posterior predictive checks were visualized with the bayesplot package (version 1.10.0; Gabry et al. 2019) before evaluating the significance of the estimated parameters in relation to our hypotheses (brms::hypothesis function). We opted for the Bayesian linear mixed model rather than the Bayesian ANCOVA (as preregistered) to increase the trustworthiness of our analysis, given that the assumptions were not fulfilled for an ANCOVA.

Descriptive data analyses were conducted in JASP 0.17.2.1 (JASP Team 2023). Log‐transformed Bayes factors were interpreted according to the Jeffrey's scheme (Goss‐Sampson 2020).

## 3 Results

### 3.1 ROI Analysis

**FIGURE 2:**
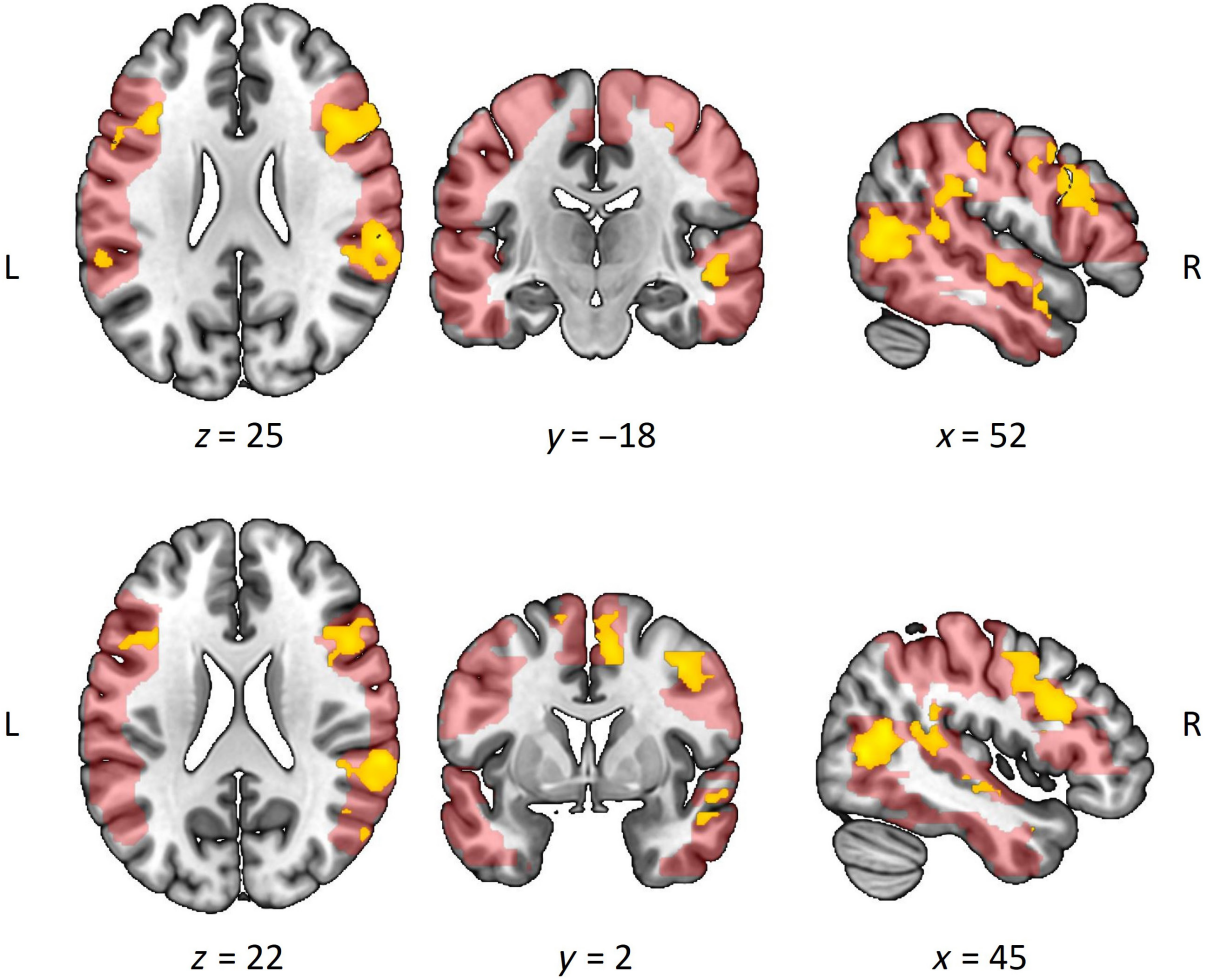
The clusters significantly modulated by observed behavioral IPS (yellow) are overlaid on the ROI mask (red) from the region of interest analysis.

Behavioral IPS as observed in the video vignettes significantly modulated activation in the lateral occipital temporal cortex (LOTC; including the LOC and MTG), IPL (SMG, AG), frontal gyri (IFG, MFG, SFG), pre‐motor areas (SMA, precentral, and postcentral gyri extending into the superior parietal lobe [SPL]), and the right superior temporal area (STG/STS) (Figure 2; Table 2), as in line with our expectation. All modulations were present in both hemispheres.

**TABLE 2:** Results of the ROI analysis. All significant clusters are reported from a non‐parametric, cluster mass extent approach considering *p* < 0.05. Coordinates are reported in MNI space.

Effect	Region	BA	H	Cluster size	*t*‐value	*x*	*y*	*z*
Non‐autistic > autistic								
	MFG	9	R	174	5.08	38	22	26
	STG	22	R	105	4.54	48	−18	−6
	MTG				3.71	68	−50	10
	AG	39	R	164	4.53	56	−46	22
Autistic > non‐autistic								
No significant clusters								
Interpersonal synchrony (IPS) modulation								
	LOC	19	R	1917	5.95	46	−68	6
	MTG	39	R		5.67	46	−58	14
	PO	40	R		5.54	62	−34	24
	SMG		R		5.23	68	−46	12
	IFG, pars op.	44	R	1245	5.43	42	16	28
	MFG	6	R		4.99	40	2	44
	Precentral gyrus	44	R		4.65	48	12	32
	LOC	19	L	389	5.28	−50	−70	8
	AG	39	L		3.89	−54	−56	14
	SMG	39	L		3.52	−54	−48	18
	MFG	9	L	171	4.93	−36	20	26
	IFG, pars op.	44	L		3.77	−46	18	20
	Precentral gyrus		L	403	4.70	−20	−12	64
	SFG		L		4.02	−16	−12	62
	SMA	6	R	500	4.68	10	2	52
	SFG	6	R		4.06	8	12	62
	SFG	6	L		3.80	−10	10	68
	STG	22	R	279	4.57	50	−16	−8
	Planum polare	22	R		4.18	50	−6	−10
	Temporal pole	38	R		4.08	48	6	−26
	Postcentral gyrus	1	R	163	4.32	32	−32	46
	SMG	40	R		3.93	40	−32	38
	SPL	40	R		3.43	40	−40	48
	Postcentral gyrus	40	L	87	4.30	−42	−36	50
	PO	40	L	92	3.83	−58	−40	26
	SMG	39	L		3.25	−48	−44	38

Abbreviations: BA: Brodmann area; H: Hemisphere; L: Left; R: Right.

In contrast to our expectation though, the effect of IPS that was observed on neural activation did not differ between autistic and non‐autistic participants.

**FIGURE 3:**
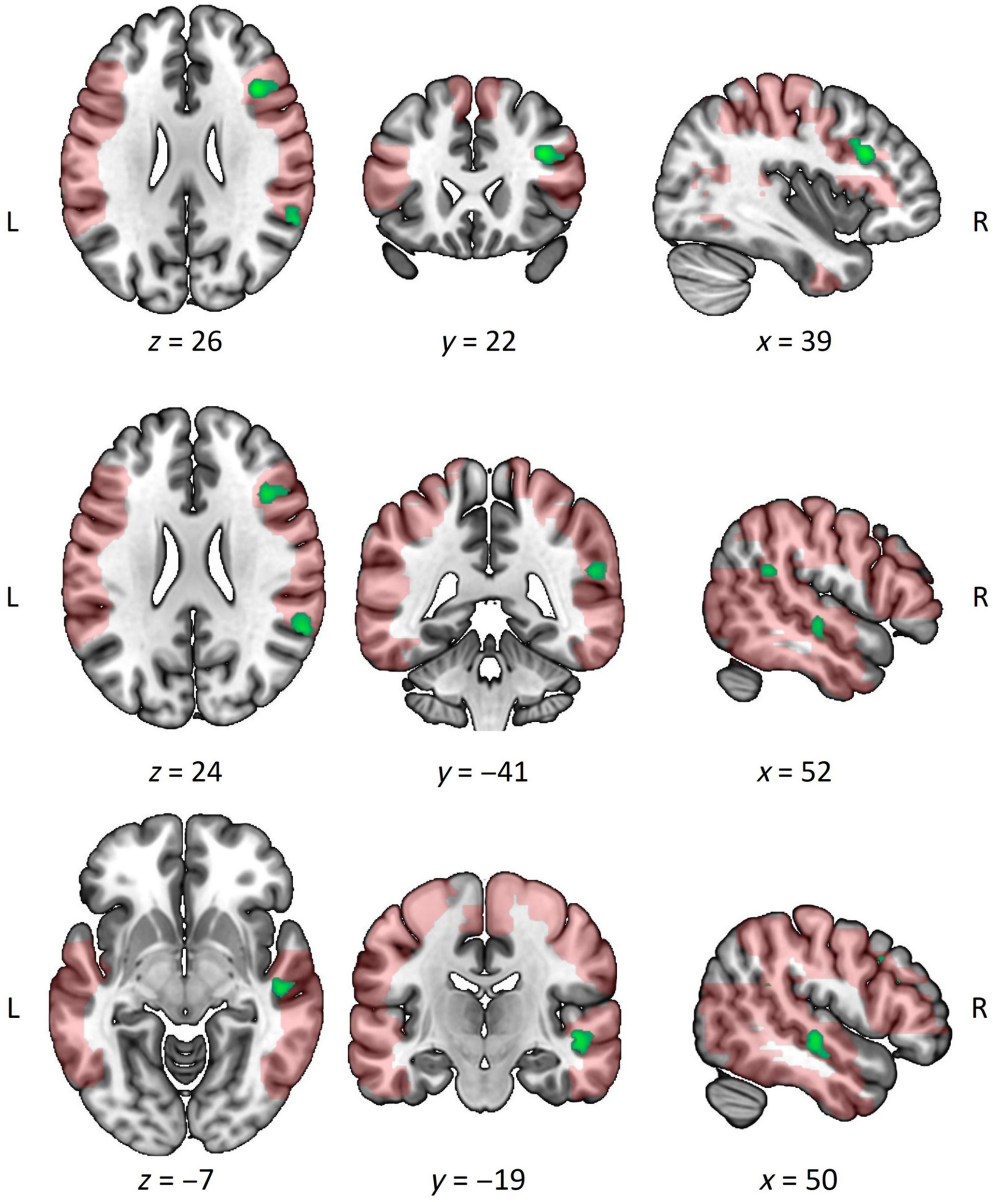
Significant clusters of increased task activation (green), as a measure of general social interaction perception, for non‐autistic compared with autistic participants are overlaid on the ROI mask (red) from the region of interest analysis. There were no significant clusters in the autistic > non‐autistic contrast.

Moreover, there was increased neural activation in the right middle frontal gyrus (MFG), angular gyrus (AG), and the STG, for non‐autistic compared with autistic participants viewing the social interactions, regardless of IPS (Figure 3; Table 2).

### 3.2 Whole Brain Analysis

**FIGURE 4:**
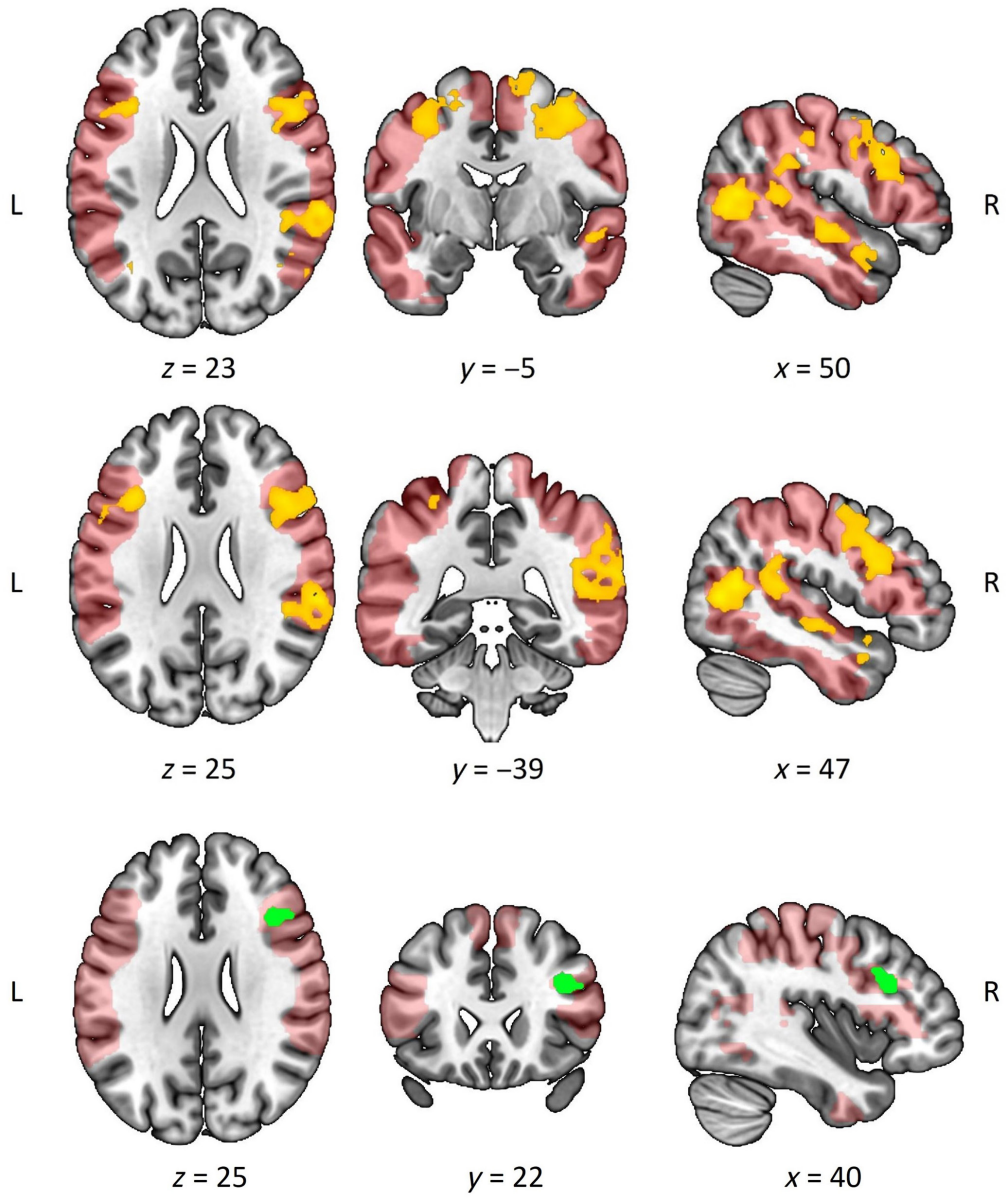
Surviving clusters from whole brain analyses. The clusters significantly modulated by behavioral IPS that were observed in the video vignettes (yellow) and increased task activation (green) for non‐autistic compared with autistic participants are overlaid on the ROI mask (red). There were no significant clusters in the autistic > non‐autistic contrast.

For IPS modulation, the whole brain analysis yielded similar activation to the ROI analysis but revealed distinct clusters of activation encompassing the bilateral SPL and additionally the parietal operculum, extending into part of the right precuneus (Figure 4; Supporting Information S7). The main effect of group demonstrated increased activation of the MFG for non‐autistic compared with autistic participants while observing social interactions. As in the ROI analysis, there was no interaction effect, wherein the neural processing of IPS in social interaction perception did not differ between autistic and non‐autistic participants. We also found no association between the neural correlates of observed IPS and the behavioral execution of IPS in the pooled sample and no differences between groups.

### 3.3 Effects of Task and Stimuli

As a sanity check, we assessed the effect of task (no modulation of behavioral IPS that was observed in the video vignettes as assessed using a pooled sample) and found activation in the expected visual processing regions including the bilateral occipital and LOC, as well as recruitment of the bilateral temporal fusiform cortex and right ITG, extending into the bilateral SMG and right AG. Additionally, there was significant activation in the bilateral IFG, right MFG and frontal pole, and right STG (Supporting Information S8).

Furthermore, as the stimuli were comprised of non‐autistic and mixed dyads (autistic/non‐autistic), we explored whether the dyad composition of the stimuli might have influenced neural activation, given that behavioral IPS has been shown to be reduced in dyads including an autistic and non‐autistic individual (Georgescu et al. 2020; Koehler et al. 2021). We found similar neural activation between the stimuli with respect to dyad composition (mixed > non‐autistic, non‐autistic > mixed), which was also comparable for autistic and non‐autistic participants; thus, suggesting that the previously reported activation was modulated by the behavioral IPS that was observed rather than dyad composition.

### 3.4 Behavioral Ratings

**FIGURE 5:**
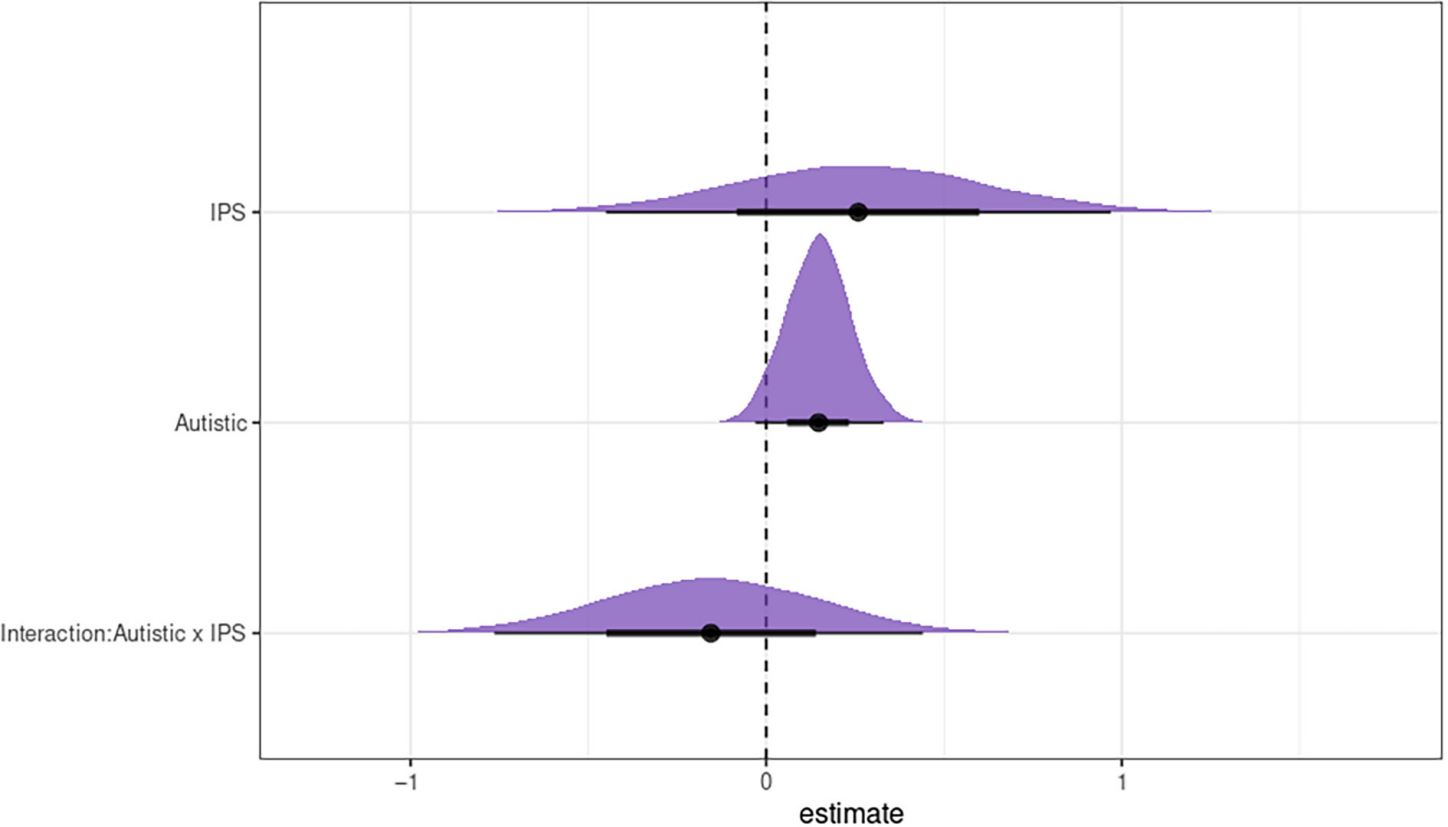
Posterior sample distributions are shown for each predictor from the Bayesian linear mixed model. The solid lines (bold, thin) reflect the 95% and 66% intervals of the distributions. Neither behavioral IPS that was observed in the video vignettes, participants' diagnostic group, nor the interaction with diagnostic group, had a credible effect on the behavioral ratings.

For the behavioral ratings used to ensure that participants actively attended to each video (Figure 5), the Bayesian mixed model revealed that the behavioral IPS that was observed had a positive effect on the ratings, although, against our expectation, the evidence was not sufficiently credible (estimate = 0.26; posterior probability = 0.76). Visually, autistic participants reported higher ratings than non‐autistic participants; however, the evidence was not sufficiently credible to support a group difference (estimate = 0.15; posterior probability = 0.94). The interaction effect of diagnostic group and observed behavioral IPS did not influence the ratings (estimate = −0.16; posterior probability = 0.31).

## 4 Discussion

How one perceives a social interaction is arguably important for how one learns to engage in social interactions. Yet, the neuro‐perceptual basis of dynamic social interaction perception has only recently been investigated, particularly regarding its temporal dynamics observed in avatar behaviors (Georgescu et al. 2014; Tsantani, Yon, and Cook 2024). Such temporal coordination of behaviors, namely behavioral IPS, is an important feature that mediates several qualities of social interactions in varying contexts, such as affiliation (e.g., Hove and Risen 2009; Miles, Nind, and Macrae 2009), cooperation (e.g., Miles et al. 2017; Wiltermuth and Heath 2009), and trust (e.g., Mitkidis et al. 2015; Tamborini et al. 2018). Establishing IPS requires perceptual processing of temporal features of interactive behaviors to allow interaction partners to adapt their behaviors in real‐time, with which autistic adults have been known to struggle (McNaughton and Redcay 2020). The aim of the current study was to evaluate the influence of temporal dynamics of social interactions on neural processing and potential differences in these neural processes in autism. For this purpose, participants observed silent video vignettes of human dyadic interactions that varied in behavioral IPS. While observing these video vignettes, neural activation in the AON was associated with the temporal dynamics of social interactions, specifically IPS. Although there was an expected group difference in the neural processing of social interactions between autistic and non‐autistic participants, no group difference was found for the neural processing of the temporal dynamics of the social interactions. Both autistic and non‐autistic participants engaged the AON for the latter aspect in a comparable way.

Behavioral IPS that was observed in the video vignettes had a modulatory effect on AON activation in the current observational fMRI paradigm. This finding echoes previous reports of increased AON activation for synchronous stimuli in virtual characters' behaviors (Georgescu et al. 2014; Tsantani, Yon, and Cook 2024). Notably, Georgescu et al. (2014) found increased activation in the superior temporal areas, LOTC, IPL, and frontal gyri, as opposed to the social neural network, for contingent movements in virtual characters' behaviors. In line with these reports from controlled virtual character paradigms, the present study adds to the converging evidence that the perception of temporal dynamics of social interactions is processed by the AON. The strongest activation was observed in the LOTC and IPL, especially in the right hemisphere. These regions are associated with action observation (Spunt, Satpute, and Lieberman 2011), inference (Kana and Travers 2012; Kuhlen et al. 2015), and imitation (Fishman et al. 2015; Krüger et al. 2014), as well as social interaction perception (Abassi and Papeo 2022; Centelles et al. 2011; Isik et al. 2017; Walbrin, Downing, and Koldewyn 2018; Walbrin and Koldewyn 2019). The IPL, particularly the SMG and AG extended into the TPJ, is well‐associated with mentalization and Theory of Mind tasks (Kliemann and Adolphs 2018; Müller and Fishman 2018; Saxe and Kanwisher 2003; Van Overwalle and Baetens 2009). This might suggest that participants drew on regions associated with mentalization even though there was no need to use or integrate this information since they were merely observing the interactions.

Moreover, activation of the frontal gyri was significantly modulated by observed behavioral IPS. These regions, particularly the IFG, are engaged during action observation, specifically of whole‐body movements (Caspers et al. 2010), action intention (Iacoboni et al. 2005; Möttönen, Farmer, and Watkins 2016), as well as motor planning and control (Fontana et al. 2012; Liakakis, Nickel, and Seitz 2011). Furthermore, we observed right superior temporal activation, including the STS, which is widely associated with biological motion processing during action observation (Caspers et al. 2010; Deen et al. 2015; Freitag et al. 2008; Kuhlen et al. 2015; Su, Culotta, Hoffman, et al. 2020) and is considered a hub for social interaction perception (Arioli and Canessa 2019; Centelles et al. 2011; Isik et al. 2017; Landsiedel et al. 2022; Tsantani, Yon, and Cook 2024; Walbrin, Downing, and Koldewyn 2018; Walbrin and Koldewyn 2019).

Recently, recruitment of the frontal gyri and STS for IPS processing have been implicated during live social interactions in autistic and non‐autistic individuals. Su and colleagues (Su, Culotta, Hoffman, et al. 2020; Su, Culotta, Mueller, et al. 2020) investigated the neural basis of action observation, action execution, and IPS processing using functional near‐infrared spectroscopy (fNIRS). They found increased activation in the right IFG region, as well as the STS region, for action execution and IPS processing during a clean‐up task when non‐autistic children interacted with a partner (Su, Culotta, Hoffman, et al. 2020). STS activation has also been implicated in non‐autistic adults when they observed avatars' head movement synchrony. Tsantani, Yon, and Cook (2024) found significant synchrony classification accuracy in the STS and EBA as well as decoding of synchrony in the middle temporal cortex when participants observed synchronous compared to asynchronous head movements. In line with these findings, the present investigation of IPS in social interaction perception when observing human social interactions also showed a modulatory effect of observed behavioral IPS on neural activation in the frontal gyri and STS, in addition to sensorimotor regions associated with dynamic motion processing and motoric planning (Sulpizio et al. 2023). Taken together, this evidence emphasizes that the LOTC, STS, and sensorimotor regions play an important role in processing spatiotemporal information, as well as the frontal gyri in action observation and planning, for processing the temporal dynamics of social interactions.

The current findings further suggest that neural processes underlying the perception of temporal dynamics of social interactions are not differentiated in autism, which logically dismisses it as a potential mechanism explaining reduced behavioral IPS. The established regions recruited for IPS perception did not show differences in activation in autistic and non‐autistic participants, indicating intact neural processing of temporal dynamics of social interactions in autism. Moreover, this rationale is further supported by our findings showing no association between the neural correlates of the temporal dynamics of social interactions and behavioral IPS execution from the same participants, despite reduced behavioral IPS execution for dyads with an autistic participant compared to dyads with a non‐autistic participant (Bierlich, Scheel, et al. 2024). Together, these findings align with a recent investigation of gesture perception in autistic adults (Fourie et al. 2020). Fourie et al. (2020) found comparable neural activation patterns in the AON, as well as behavioral gesture recognition, between autistic and non‐autistic individuals when they observed functional and communicative gestures by an avatar. However, the authors also report differentiated bilateral processing patterns in the STS for gesture intensity for autistic versus non‐autistic participants, possibly related to differences in motion processing (Fourie et al. 2020). Similarly, we captured the perceptual basis of social interactions when participants *observed* an interaction, which elicited comparable activation patterns between autistic and non‐autistic participants as modulated by the behavioral IPS that they observed.

In the broader context of action perception in autism, our findings diverge with a recent meta‐analysis demonstrating that altered low‐level temporal features may be indicative of attenuated action perception in autism (Federici et al. 2020). Notably though, the included studies were limited to behavioral detection of biological motion in point‐light displays. Additionally, those studies that specifically focus on the temporal dynamics comprised a small sample (*n* = 6), of which half found intact action perception in autism (Burnside, Wright, and Poulin‐Dubois 2017; Cusack, Williams, and Neri 2015; von der Lühe et al. 2016). Action perception is inherently implicated in *social interaction perception*, as one observes the actions of others. Our findings are in line with literature showing intact action perception of temporal dynamics and extend this at the neural level to intact perception of IPS in social interactions in autism when observing human dyadic social interactions. Thus, there is converging evidence against altered neural processing of temporal dynamics of social interactions in autism, indicating that this aspect does not fully account for attenuated behavioral IPS in autism.

Presently, we speculate that attenuated behavioral IPS in autism may rather be based on difficulties with mentalization and top‐down modulation from prefrontal regions when one is *part of* an interaction and must actively use and temporally coordinate nonverbal behaviors to communicate. Su, Culotta, Mueller, et al. (2020) found decreased IFG and MFG, as well as MTG and STG, activation of IPS processing for autistic individuals using an interactive clean‐up task with autistic children. Such differential activation patterns contrast the present study. Importantly though, Su, Culotta, Mueller, et al. (2020) assessed the behavioral production and neural activation of IPS processes when individuals *engaged in* a social interaction. This supports the idea that one's role in an interaction may be critical for how IPS is processed at the neural level and is in line with the additive approach for investigating social interactions (Hamilton and Holler 2023). Hyperscanning studies may further shed light onto the mechanisms of neural (i.e., brain‐to‐brain) IPS in autistic individuals and their interaction partners when they are engaged in live social interactions, with initial evidence demonstrating differentiated inter‐brain synchrony for dyads including an autistic individual when observing and imitating their partners (Moreau et al. 2024).

More broadly, some neuroimaging evidence has shown atypical processing of social cognition and mentalization in autism (Andreou and Skrimpa 2020; Müller and Fishman 2018; Sato and Uono 2019; Velikonja, Fett, and Velthorst 2019; but see also Moessnang et al. 2020); processes that are often linked to prefrontal activation. Moreover, altered mimicry—the imitation of another person's behavior—has been shown in autism in accordance with the social top‐down response modulation (STORM) model (Wang and Hamilton 2012). This account posits that mimicry is socially driven by top‐down mechanisms processed by the prefrontal cortex, which may be atypical in autism (Hamilton 2015; Wang and Hamilton 2012). The present findings partly support the STORM model of mimicry (Wang and Hamilton 2012) and could extend this to IPS perception in future investigations. Thus, our findings may hint that the integration of social top‐down expectations with perceived information for one's own action planning and execution during social interactions could potentially explain attenuated behavioral IPS found in autism.

While neuro‐perceptual processing of temporal dynamics of behavior might be spared, the *use* of the perceived information for dynamic mutual adjustment of behaviors during social interactions could be the locus of difficulty (see also the conclusions by Cusack, Williams, and Neri 2015). When one is part of an interaction, one must incorporate the perceived information from an interaction partner and dynamically update one's own expectations, for instance, in order to predict the next turn. This integration of the information may have implications in line with predictive coding accounts of autism (Haker, Schneebeli, and Stephan 2016). Indeed, von der Lühe et al. (2016) found reduced interpersonal action prediction, but not action perception, for autistic individuals and related these findings to altered predictive coding in autism. Although, other studies centered around predictive coding accounts of autism suggest that the use of perceived information does not differ for autistic individuals but may rather differ in their expectations (e.g., Robles et al. 2024) or delayed updating of their expectations (e.g., Vishne et al. 2021). As such, predictive coding may be an informative lens for future studies to apply to the processing of social interactions in order to further disentangle the underlying mechanisms.

Furthermore, our results demonstrate differences in general social interaction perception in autism. There was increased neural activation in the right MFG, STG/STS, and AG for non‐autistic compared with autistic participants when observing social interactions. This finding partially aligns with the literature regarding differentiated AON activation for social cognition in autism (Chan and Han 2020; Kilroy, Cermak, and Aziz‐Zadeh 2019; Pokorny et al. 2018; Yang and Hofmann 2016). Notably, differences presently observed in neural mechanisms of social interaction perception between autistic and non‐autistic individuals were not due to differences in processing the temporal dynamics of these social interactions as neural activation patterns were comparable when modulated by IPS in observed dyads. The present task also captured various processes involved in social interaction perception (e.g., action observation, action planning, action inference), irrespective of the temporal dynamics of social interactions, suggesting that other social cognitive processes may be perceived differently by autistic individuals. As follows, we speculate about potential explanations for the presently observed differences.

One possible explanation could be related to atypical action observation and planning processes. Neuroimaging evidence demonstrates reduced activation in the frontal gyri and temporal–parietal regions for biological motion perception in autism (e.g., Freitag et al. 2008; Koldewyn, Whitney, and Rivera 2011; for a review see, Todorova, Hatton, and Pollick 2019). Moreover, reduced activation of the MFG in autistic individuals has been linked to executive function difficulties, including planning and motor‐related inhibition (Ikeda et al. 2018; for a review, see May and Kana 2020). As such, differences in MFG activation in the present study may hint at difficulties related to higher‐order action planning for autistic participants. Together, differences in action observation, which our findings show was irrespective of the temporal dynamics, and planning process could be reflected by the differentiated activation in the frontal gyri and temporal–parietal regions for social interaction perception that was presently observed.

Another possibility underlying the differentiated activation of general social interaction perception could involve altered mentalizing processes. Temporal–parietal regions, namely the STS and TPJ, are engaged in implicit and explicit mentalization (Frith and Frith 2021; Schneider et al. 2014). Reduced neural activation of temporal‐partial areas has further been reported for implicit and explicit mentalization in autistic individuals, despite similar behavioral performance compared to non‐autistic individuals (Nijhof et al. 2018). Although explicit mentalization was not required for participants to engage in the present task, it is arguably possible that non‐autistic participants may have implicitly mentalized about the dyads they observed. If so, differences in implicit mentalization processes could possibly explain the reduced activation we observed in the STS/STG and AG for autistic participants, who arguably might have engaged less in implicit mentalization.

A third speculation could be that autistic and non‐autistic participants approached the task differently, possibly reflecting differences in attentional processes during the task. Previous findings report reduced fixation durations and latencies, as well as differentiated pupillary responses, during social scene perception for autistic individuals (Chevallier et al. 2015; Frost‐Karlsson et al. 2019; Rigby, Stoesz, and Jakobson 2016). This could reflect differences in attention allocation during social scene processing. To discern whether the observed differentiated processing results from other processes involved in social interaction perception or differences in attention allocation, future studies may consider including eye‐tracking to assess individuals' gaze and fixation patterns, as well as neuropsychological evaluations of attention.

Finally, there are a few considerations regarding the stimulus set to note. Importantly, the stimulus set used in the current paradigm allowed us to capture aspects related to the temporal dynamics of movements by omitting other socially relevant factors (e.g., audio, facial features) in a purely observational task. This required participants to actively view videos of social interactions with varying amounts of behavioral IPS extracted from the dyads in the videos. It could be that observing more complex social situations (e.g., triadic interactions) may elicit differences between the groups. The behavioral check, which purely served the purpose of assuring attention to the vignettes, indeed confirmed that participants actively attended to the task in a similar manner. Behavioral IPS extracted from the stimuli did not influence ratings of the scene, nor did the ratings credibly differ between groups. Furthermore, one caveat to consider is the role of the dyad composition in the observed video vignettes with regard to the Double Empathy hypothesis (Milton 2012). Some studies have found increased feelings of rapport (Crompton, Ropar, et al. 2020; Crompton, Sharp, et al. 2020; Morrison et al. 2020) and observations of rapport (Crompton, Sharp, et al. 2020; Jones et al. 2024) for dyads consisting of two non‐autistic individuals or two autistic individuals, compared with dyads including an autistic and a non‐autistic individual. More specifically regarding the temporal dynamics of social interactions, Georgescu et al. (2020) found reduced behavioral IPS in dyads including an autistic individual, regardless of the diagnostic status of the interaction partner. Their findings are further supported by evidence demonstrating increased variability in intra‐individual timing during communication in autism, which lends to increased decoding costs for the observing interaction partner (Bloch et al. 2022, 2024). These decoding costs were higher for autistic individuals when observing the autistic communication style, suggesting a potential disadvantage when an autistic person decodes an autistic communication style (Bloch et al. 2023, 2024). To assess whether the dyad composition (homogenous vs. heterogenous) was processed differently by autistic and non‐autistic participants, the exploratory whole brain analysis of dyad composition from the observed interactions showed neither a main effect for neural activation nor an interaction effect with the diagnostic status of observers. As such, we can be confident that IPS variation observed in the interactions modulated neural activity rather than other features, such as diagnostic status of the depicted interactants.

## 5 Conclusion

The present study demonstrated that behavioral IPS modulates regions of the AON when observing human dyadic interactions. We further showed that these processing patterns are spared in autism and were not associated with behavioral IPS produced by the same participants when they interacted with another person. Thus, altered neural processing of temporal dynamics in social interactions does not account for attenuated behavioral IPS in autism. Nevertheless, there were differences in neural activation patterns for autistic individuals during general social interaction perception. Together, these findings demonstrate differentiated neural processing of social interactions between autistic and non‐autistic participants, and that the temporal dynamics of these social interactions were not the cause thereof. In conclusion, spared AON engagement for the neural processing of temporal dynamics of social interactions in autism render this aspect unlikely to account (i) for the widely reported and presently observed attenuation of IPS in autism and (ii) for widely reported and presently observed differences in social interaction perception in autism.

## Author Contributions

**A.M.B.:** methodology, software, project administration, investigation, data curation, formal analysis, visualization, writing – original draft, writing – review and editing. **N.T.S.:** investigation, writing – review and editing. **L.S.T.:** methodology, writing – review and editing. **D.K.:** data curation, supervision, writing – review and editing. **R.T.:** methodology, writing – review and editing. **A.L.G.:** methodology, writing – review and editing. **J.C.K.:** methodology, writing – review and editing. **I.S.P.:** methodology, supervision, writing – review and editing. **C.M.F.‐W.:** conceptualization, funding acquisition, resources, supervision, writing – review and editing. **I.S.P.** and **C.M.F.‐W.** share last authorship.

## Ethics Statement

Participants provided informed consent, in accordance with the Declaration of Helsinki (World Medical Association 2013), to participate in the study. This study was approved by the ethics committee of the Medical Faculty at LMU Munich (No. 20‐1050).

## Conflicts of Interest

The authors declare no conflicts of interest.

## Supporting information


**Data S1.** Supporting Information.

## Data Availability

A preregistration, scripts, preprocessed behavioral ratings, and statistical maps are available on OSF (https://osf.io/cw7n4). The full dataset underlying this article may be shared after anonymization upon reasonable request to the corresponding authors (A.M.B, C.M.F.‐W.).
