# Supplementary material for: Neural Mechanisms of Social Interaction Perception: Observing Interpersonal Synchrony Modulates Action Observation Network Activation and Is Spared in Autism
Source: Hum Brain Mapp. 2024 Oct 24;45(15):e70052. doi: 10.1002/hbm.70052 (PMC11502411; doi:10.1002/hbm.70052)

**Neural mechanisms of social interaction perception: Observing interpersonal synchrony modulates Action Observation Network activation and is spared in autism**

**Bierlich, A.M., Scheel, N.T., Traiger, L.S., Keeser, D., Tepest, R., Georgescu, A.L., Koehler, J.C., Plank, I.S.** **^†^ & Falter-Wagner, C.M. ^†^**

^†^  These authors share last authorship.

# Supplementary Material

## S1. Behavioral interpersonal synchrony (IPS) of the stimulus set

**Figure S1.1.** The raw behavioral IPS values are shown across of the stimulus set. Centered behavioral IPS were used as a parametric modulator of interest to evaluate the main effect of IPS on neural activation in the fMRI analysis.


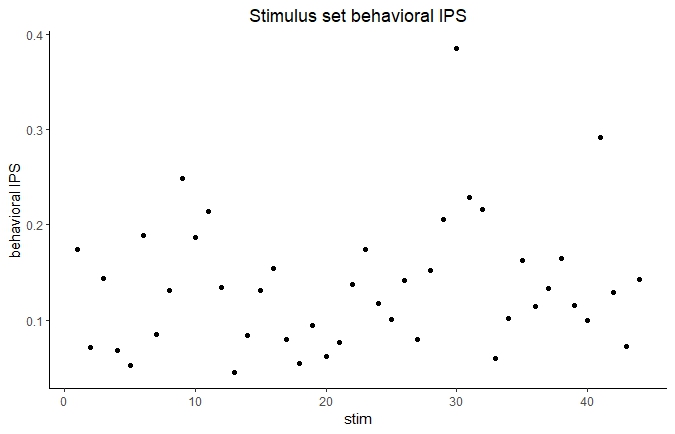


## S2. *fMRIPrep* Pre-processing Pipeline

Results included in this manuscript come from preprocessing performed using fMRIPrep 22.1.1 (Esteban, Markiewicz, et al. (2018); Esteban, Blair, et al. (2018); RRID:SCR_016216), which is based on Nipype 1.8.5 (K. Gorgolewski et al. (2011); K. J. Gorgolewski et al. (2018); RRID:SCR_002502).

#### Preprocessing of B0 inhomogeneity mappings

A total of 2 fieldmaps were found available within the input BIDS structure for this particular subject. A B0-nonuniformity map (or fieldmap) was estimated based on two (or more) echo-planar imaging (EPI) references with topup (Andersson, Skare, and Ashburner (2003); FSL 6.0.5.1:57b01774).

#### Anatomical data preprocessing

A total of 1 T1-weighted (T1w) images were found within the input BIDS dataset.The T1-weighted (T1w) image was corrected for intensity non-uniformity (INU) with N4BiasFieldCorrection (Tustison et al. 2010), distributed with ANTs 2.3.3 (Avants et al. 2008, RRID:SCR_004757), and used as T1w-reference throughout the workflow. The T1w-reference was then skull-stripped with a Nipype implementation of the antsBrainExtraction.sh workflow (from ANTs), using OASIS30ANTs as target template. Brain tissue segmentation of cerebrospinal fluid (CSF), white-matter (WM) and gray-matter (GM) was performed on the brain-extracted T1w using fast (FSL 6.0.5.1:57b01774, RRID:SCR_002823, Zhang, Brady, and Smith 2001). Brain surfaces were reconstructed using recon-all (FreeSurfer 7.2.0, RRID:SCR_001847, Dale, Fischl, and Sereno 1999), and the brain mask estimated previously was refined with a custom variation of the method to reconcile ANTs-derived and FreeSurfer-derived segmentations of the cortical gray-matter of Mindboggle (RRID:SCR_002438, Klein et al. 2017). Volume-based spatial normalization to two standard spaces (MNI152NLin6Asym, MNI152NLin2009cAsym) was performed through nonlinear registration with antsRegistration (ANTs 2.3.3), using brain-extracted versions of both T1w reference and the T1w template. The following templates were selected for spatial normalization: FSL’s MNI ICBM 152 non-linear 6th Generation Asymmetric Average Brain Stereotaxic Registration Model [Evans et al. (2012), RRID:SCR_002823; TemplateFlow ID: MNI152NLin6Asym], ICBM 152 Nonlinear Asymmetrical template version 2009c [Fonov et al. (2009), RRID:SCR_008796; TemplateFlow ID: MNI152NLin2009cAsym].

#### Functional data preprocessing

For each of the 2 BOLD runs found per subject (across all tasks and sessions), the following preprocessing was performed. First, a reference volume and its skull-stripped version were generated using a custom methodology of fMRIPrep. Head-motion parameters with respect to the BOLD reference (transformation matrices, and six corresponding rotation and translation parameters) are estimated before any spatiotemporal filtering using mcflirt (FSL 6.0.5.1:57b01774, Jenkinson et al. 2002). The estimated fieldmap was then aligned with rigid-registration to the target EPI (echo-planar imaging) reference run. The field coefficients were mapped on to the reference EPI using the transform. BOLD runs were slice-time corrected to 0.991s (0.5 of slice acquisition range 0s-1.98s) using 3dTshift from AFNI (Cox and Hyde 1997, RRID:SCR_005927). The BOLD reference was then co-registered to the T1w reference using bbregister (FreeSurfer) which implements boundary-based registration (Greve and Fischl 2009). Co-registration was configured with six degrees of freedom. Several confounding time-series were calculated based on the preprocessed BOLD: framewise displacement (FD), DVARS and three region-wise global signals. FD was computed using two formulations following Power (absolute sum of relative motions, Power et al. (2014)) and Jenkinson (relative root mean square displacement between affines, Jenkinson et al. (2002)). FD and DVARS are calculated for each functional run, both using their implementations in Nipype (following the definitions by Power et al. 2014). The three global signals are extracted within the CSF, the WM, and the whole-brain masks. Additionally, a set of physiological regressors were extracted to allow for component-based noise correction (CompCor, Behzadi et al. 2007). Principal components are estimated after high-pass filtering the preprocessed BOLD time-series (using a discrete cosine filter with 128s cut-off) for the two CompCor variants: temporal (tCompCor) and anatomical (aCompCor). tCompCor components are then calculated from the top 2% variable voxels within the brain mask. For aCompCor, three probabilistic masks (CSF, WM and combined CSF+WM) are generated in anatomical space. The implementation differs from that of Behzadi et al. in that instead of eroding the masks by 2 pixels on BOLD space, a mask of pixels that likely contain a volume fraction of GM is subtracted from the aCompCor masks. This mask is obtained by dilating a GM mask extracted from the FreeSurfer’s aseg segmentation, and it ensures components are not extracted from voxels containing a minimal fraction of GM. Finally, these masks are resampled into BOLD space and binarized by thresholding at 0.99 (as in the original implementation). Components are also calculated separately within the WM and CSF masks. For each CompCor decomposition, the k components with the largest singular values are retained, such that the retained components’ time series are sufficient to explain 50 percent of variance across the nuisance mask (CSF, WM, combined, or temporal). The remaining components are dropped from consideration. The head-motion estimates calculated in the correction step were also placed within the corresponding confounds file. The confound time series derived from head motion estimates and global signals were expanded with the inclusion of temporal derivatives and quadratic terms for each (Satterthwaite et al. 2013). Frames that exceeded a threshold of 0.5 mm FD or 1.5 standardized DVARS were annotated as motion outliers. Additional nuisance timeseries are calculated by means of principal components analysis of the signal found within a thin band (crown) of voxels around the edge of the brain, as proposed by (Patriat, Reynolds, and Birn 2017). The BOLD time-series were resampled into standard space, generating a preprocessed BOLD run in MNI152NLin6Asym space. First, a reference volume and its skull-stripped version were generated using a custom methodology of fMRIPrep. Automatic removal of motion artifacts using independent component analysis (ICA-AROMA, Pruim et al. 2015) was performed on the preprocessed BOLD on MNI space time-series after removal of non-steady state volumes and spatial smoothing with an isotropic, Gaussian kernel of 6mm FWHM (full-width half-maximum). Corresponding “non-aggresively” denoised runs were produced after such smoothing. Additionally, the “aggressive” noise-regressors were collected and placed in the corresponding confounds file. All resamplings can be performed with a single interpolation step by composing all the pertinent transformations (i.e. head-motion transform matrices, susceptibility distortion correction when available, and co-registrations to anatomical and output spaces). Gridded (volumetric) resamplings were performed using antsApplyTransforms (ANTs), configured with Lanczos interpolation to minimize the smoothing effects of other kernels (Lanczos 1964). Non-gridded (surface) resamplings were performed using mri_vol2surf (FreeSurfer).

Many internal operations of fMRIPrep use Nilearn 0.9.1 (Abraham et al. 2014, RRID:SCR_001362), mostly within the functional processing workflow. For more details of the pipeline, see the section corresponding to workflows in fMRIPrep’s documentation.

#### Copyright Waiver

The above boilerplate text was automatically generated by fMRIPrep with the express intention that users should copy and paste this text into their manuscripts unchanged. It is released under the CC0 license.

#### References

Abraham, Alexandre, Fabian Pedregosa, Michael Eickenberg, Philippe Gervais, Andreas Mueller, Jean Kossaifi, Alexandre Gramfort, Bertrand Thirion, and Gael Varoquaux. 2014. “Machine Learning for Neuroimaging with Scikit-Learn.” Frontiers in Neuroinformatics 8. https://doi.org/10.3389/fninf.2014.00014.

Andersson, Jesper L. R., Stefan Skare, and John Ashburner. 2003. “How to Correct Susceptibility Distortions in Spin-Echo Echo-Planar Images: Application to Diffusion Tensor Imaging.” NeuroImage 20 (2): 870–88. https://doi.org/10.1016/S1053-8119(03)00336-7.

Avants, B. B., C. L. Epstein, M. Grossman, and J. C. Gee. 2008. “Symmetric Diffeomorphic Image Registration with Cross-Correlation: Evaluating Automated Labeling of Elderly and Neurodegenerative Brain.” Medical Image Analysis 12 (1): 26–41. https://doi.org/10.1016/j.media.2007.06.004.

Behzadi, Yashar, Khaled Restom, Joy Liau, and Thomas T. Liu. 2007. “A Component Based Noise Correction Method (CompCor) for BOLD and Perfusion Based fMRI.” NeuroImage 37 (1): 90–101. https://doi.org/10.1016/j.neuroimage.2007.04.042.

Cox, Robert W., and James S. Hyde. 1997. “Software Tools for Analysis and Visualization of fMRI Data.” NMR in Biomedicine 10 (4-5): 171–78. https://doi.org/10.1002/(SICI)1099-1492(199706/08)10:4/5<171::AID-NBM453>3.0.CO;2-L.

Dale, Anders M., Bruce Fischl, and Martin I. Sereno. 1999. “Cortical Surface-Based Analysis: I. Segmentation and Surface Reconstruction.” NeuroImage 9 (2): 179–94. https://doi.org/10.1006/nimg.1998.0395.

Esteban, Oscar, Ross Blair, Christopher J. Markiewicz, Shoshana L. Berleant, Craig Moodie, Feilong Ma, Ayse Ilkay Isik, et al. 2018. “fMRIPrep 22.1.1.” Software. https://doi.org/10.5281/zenodo.852659.

Esteban, Oscar, Christopher Markiewicz, Ross W Blair, Craig Moodie, Ayse Ilkay Isik, Asier Erramuzpe Aliaga, James Kent, et al. 2018. “fMRIPrep: A Robust Preprocessing Pipeline for Functional MRI.” Nature Methods. https://doi.org/10.1038/s41592-018-0235-4.

Evans, AC, AL Janke, DL Collins, and S Baillet. 2012. “Brain Templates and Atlases.” NeuroImage 62 (2): 911–22. https://doi.org/10.1016/j.neuroimage.2012.01.024.

Fonov, VS, AC Evans, RC McKinstry, CR Almli, and DL Collins. 2009. “Unbiased Nonlinear Average Age-Appropriate Brain Templates from Birth to Adulthood.” NeuroImage 47, Supplement 1: S102. https://doi.org/10.1016/S1053-8119(09)70884-5.

Gorgolewski, K., C. D. Burns, C. Madison, D. Clark, Y. O. Halchenko, M. L. Waskom, and S. Ghosh. 2011. “Nipype: A Flexible, Lightweight and Extensible Neuroimaging Data Processing Framework in Python.” Frontiers in Neuroinformatics 5: 13. https://doi.org/10.3389/fninf.2011.00013.

Gorgolewski, Krzysztof J., Oscar Esteban, Christopher J. Markiewicz, Erik Ziegler, David Gage Ellis, Michael Philipp Notter, Dorota Jarecka, et al. 2018. “Nipype.” Software. https://doi.org/10.5281/zenodo.596855.

Greve, Douglas N, and Bruce Fischl. 2009. “Accurate and Robust Brain Image Alignment Using Boundary-Based Registration.” NeuroImage 48 (1): 63–72. https://doi.org/10.1016/j.neuroimage.2009.06.060.

Jenkinson, Mark, Peter Bannister, Michael Brady, and Stephen Smith. 2002. “Improved Optimization for the Robust and Accurate Linear Registration and Motion Correction of Brain Images.” NeuroImage 17 (2): 825–41. https://doi.org/10.1006/nimg.2002.1132.

Klein, Arno, Satrajit S. Ghosh, Forrest S. Bao, Joachim Giard, Yrjö Häme, Eliezer Stavsky, Noah Lee, et al. 2017. “Mindboggling Morphometry of Human Brains.” PLOS Computational Biology 13 (2): e1005350. https://doi.org/10.1371/journal.pcbi.1005350.

Lanczos, C. 1964. “Evaluation of Noisy Data.” Journal of the Society for Industrial and Applied Mathematics Series B Numerical Analysis 1 (1): 76–85. https://doi.org/10.1137/0701007.

Patriat, Rémi, Richard C. Reynolds, and Rasmus M. Birn. 2017. “An Improved Model of Motion-Related Signal Changes in fMRI.” NeuroImage 144, Part A (January): 74–82. https://doi.org/10.1016/j.neuroimage.2016.08.051.

Power, Jonathan D., Anish Mitra, Timothy O. Laumann, Abraham Z. Snyder, Bradley L. Schlaggar, and Steven E. Petersen. 2014. “Methods to Detect, Characterize, and Remove Motion Artifact in Resting State fMRI.” NeuroImage 84 (Supplement C): 320–41. https://doi.org/10.1016/j.neuroimage.2013.08.048.

Pruim, Raimon H. R., Maarten Mennes, Daan van Rooij, Alberto Llera, Jan K. Buitelaar, and Christian F. Beckmann. 2015. “ICA-AROMA: A Robust ICA-Based Strategy for Removing Motion Artifacts from fMRI Data.” NeuroImage 112 (Supplement C): 267–77. https://doi.org/10.1016/j.neuroimage.2015.02.064.

Satterthwaite, Theodore D., Mark A. Elliott, Raphael T. Gerraty, Kosha Ruparel, James Loughead, Monica E. Calkins, Simon B. Eickhoff, et al. 2013. “An improved framework for confound regression and filtering for control of motion artifact in the preprocessing of resting-state functional connectivity data.” NeuroImage 64 (1): 240–56. https://doi.org/10.1016/j.neuroimage.2012.08.052.

Tustison, N. J., B. B. Avants, P. A. Cook, Y. Zheng, A. Egan, P. A. Yushkevich, and J. C. Gee. 2010. “N4itk: Improved N3 Bias Correction.” IEEE Transactions on Medical Imaging 29 (6): 1310–20. https://doi.org/10.1109/TMI.2010.2046908.

Zhang, Y., M. Brady, and S. Smith. 2001. “Segmentation of Brain MR Images Through a Hidden Markov Random Field Model and the Expectation-Maximization Algorithm.” IEEE Transactions on Medical Imaging 20 (1): 45–57. <https://doi.org/10.1109/42.906424>.

## S3. ROI mask

**Figure S3.1.** **The ROI mask used in the present study. The ROI mask includes the** bilateral IFG, superior temporal gyrus (STG), IPL and supramarginal gyrus (SMG), middle temporal gyrus (MTG), inferior temporal gyrus (ITG), as well as the supplementary motor area (SMA) and the precentral and postcentral gyri.

##
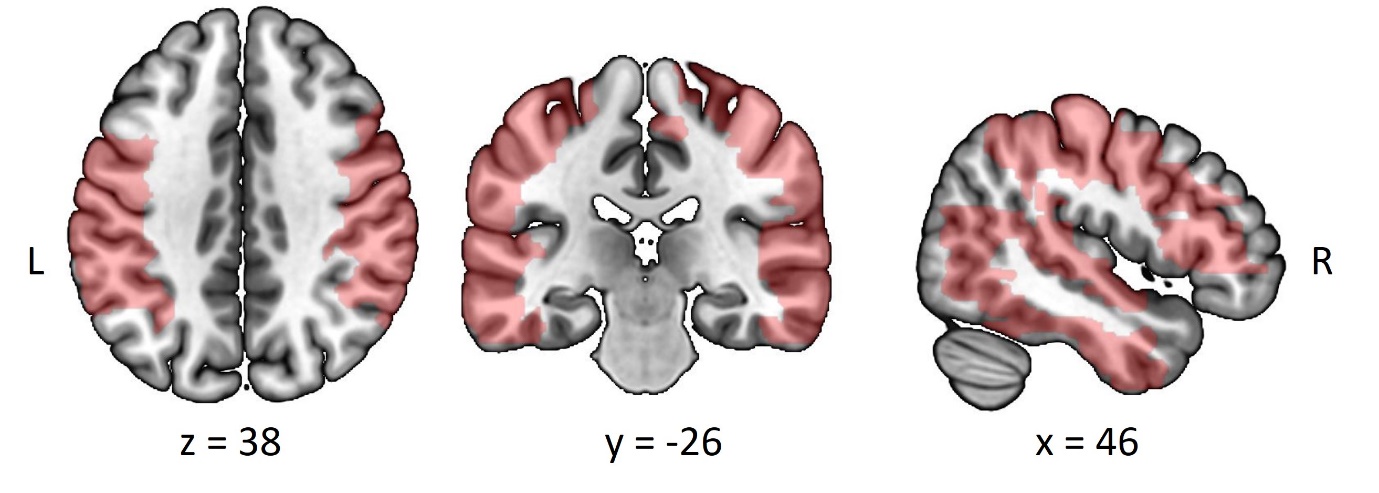


## S4. Cluster Leakage using TFCE

Initially, we planned to use TFCE thresholding with non-parametric testing as it has been reported to be more spatially sensitive and better control for false positives (Eklund et al., 2016; Smith & Nichols, 2009). However, our results indicated an effect of cluster leakage (Spisák et al., 2019) as indicated by extremely large clusters of significant activation. Upon extensive sanity checks (e.g., visual inspection of subject-level findings, verification in other statistic software, etc.), the leakage effect was only observed with TFCE (Table S4.1).

**Table S4.1.** A cluster table demonstrating a single surviving cluster from the whole brain analysis using TFCE, demonstrating cluster leakage.

| **Cluster Index** | **Voxels** | **MAX** | **MAX X (vox)** | **MAX Y (vox)** | **MAX Z (vox)** | **COG X (vox)** | **COG Y (vox)** | **COG Z (vox)** |
| --- | --- | --- | --- | --- | --- | --- | --- | --- |
| 3 | 38018 | 6.29 | 25 | 26 | 38 | 48.6 | 50.8 | 52.5 |
| 2 | 57 | 4.49 | 59 | 69 | 42 | 59 | 69.3 | 43.2 |
| 1 | 6 | 3.23 | 21 | 45 | 26 | 21.7 | 45.5 | 26.3 |

Eklund, A., Nichols, T. E., & Knutsson, H. (2016). Cluster failure: Why fMRI inferences for spatial

extent have inflated false-positive rates. Proceedings of the National Academy of Sciences of the United States of America, 113(28), 7900–7905. https://doi.org/10.1073/pnas.1602413113

Smith, S. M., & Nichols, T. E. (2009). Threshold-Free Cluster Enhancement : Addressing problems of

smoothing , threshold dependence and localisation in cluster inference FMRIB Technical Report TR08SS1. FMRIB Technical Report, 1, 1–20. https://www.fmrib.ox.ac.uk/datasets/techrep/tr08ss1/tr08ss1.pdf

Spisák, T., Spisák, Z., Zunhammer, M., Bingel, U., Smith, S., Nichols, T., & Kincses, T. (2019).

Probabilistic TFCE: A generalized combination of cluster size and voxel intensity to increase statistical power. Neuroimage, 185, 12-26.

## S5. MRIqc metrics

Select metrics depict the quality of the neuroimaging data, as produced by MRIqc. Quality metrics of the functional data (Figure S5.1) that are reported include: DVARS rate of change of BOLD signal across each data frame (dvars_nstd), DVARS normalized with the standard deviation of the temporal difference timeseries (dvars_std), DVARS normalized with voxel-wise standard deviation of the timeseries before the temporal derivative (dvars_vstd), mean framewise displacement (fd_mean), number of timepoints above framewise displacement threshold (fd_num), percentage of timepoints above framewise displacement threshold (fd_perc), temporal signal-to-noise ratio (tSNR). Quality metrics of the structural data (Figure S5.2) that are reported include: contrast-to-noise ratio (cnr), ICV fractions of the cerebral spinal fluid (icvs_csf), ICV fractions of the grey matter (icvs_gm), ICV fractions of the white matter (icvs_wm), signal-to-noise ratio (snr).

Esteban O, Birman D, Schaer M, Koyejo OO, Poldrack RA, Gorgolewski KJ; *MRIQC: Advancing*

*the Automatic Prediction of Image Quality in MRI from Unseen Sites*; PLOS ONE 12(9):e0184661; doi:[10.1371/journal.pone.0184661](https://doi.org/10.1371/journal.pone.0184661).


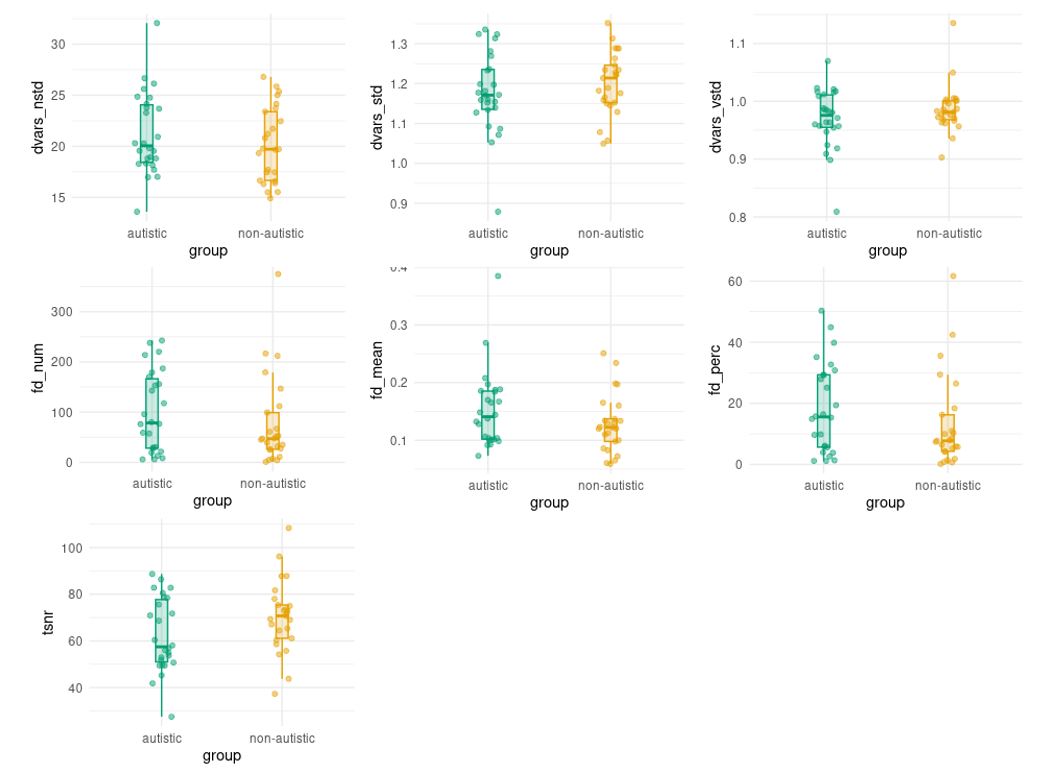
**Figure S5.1.** Panels depict group comparisons of select functional quality metrics from MRIqc.


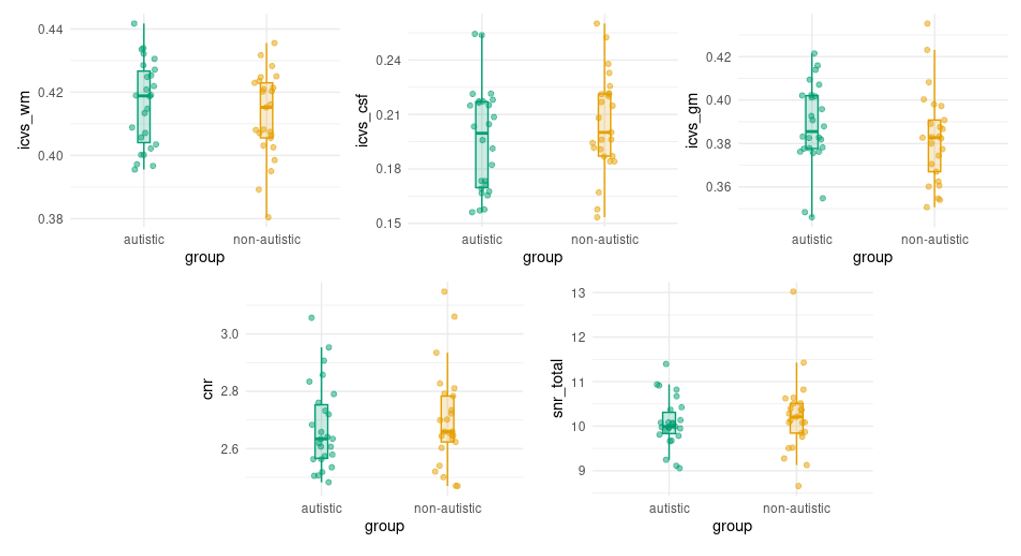
**Figure S5.2.** Panels depict group comparisons of select structural quality metrics from MRIqc.

## S6. Structural MRI comparisons

Exploratory structural analyses were conducted to compare the autistic and non-autistic groups. Using the NAMNIs pipeline (Karali et al., 2021), structural data were processed with the Juelich Brain Atlas. The intracranial volume (ICV) corrected values of grey matter and white matter for each region were compared using unpaired t-tests. No regions significantly differed, following multiple comparisons (FDR correction). Supplementary data files are available on OSF: https://osf.io/cw7n4/.

Karali, T., Padberg, F., Kirsch, V., Stoecklein, S., Falkai, P., & Keeser, D. (2021). NAMNIs:

Neuromodulation And Multimodal NeuroImaging software (0.3). Zenodo. <https://doi.org/10.5281/zenodo.4547552>

## S7. fMRI Results: Exploratory whole brain analysis

The significant clusters from the exploratory whole brain analysis of IPS is reported in Table S7.1. The corresponding figure is reported in the main manuscript.

**Table S7.1.** Results of the whole brain analysis. All significant clusters are reported from a non-parametric, cluster mass extent approach considering p < .05. Coordinates are reported in MNI space. BA: Brodmann Area; H: Hemisphere; L: Left; R: Right

| Effect | Region | BA | H | Cluster Size | t-value | x | y | z |
| --- | --- | --- | --- | --- | --- | --- | --- | --- |
| Non-autistic > Autistic | | | | | | | | |
|  | **MFG** | 9 | R | 189 | 5.08 | 38 | 22 | 26 |
| Interpersonal Synchrony (IPS) | | | | | | | | |
|  | **LOC** | 19 | L | 1031 | 6.29 | -40 | -74 | 4 |
|  | MTG | 39 | L |  | 4.14 | -46 | -62 | 12 |
|  | AG | 39 | L |  | 3.9 | -54 | -56 | 14 |
|  | **LOC** | 19 | R | 2142 | 5.95 | 46 | -68 | 6 |
|  | MTG | 39 | R |  | 5.67 | 46 | -58 | 14 |
|  | PO | 40 | R |  | 5.54 | 62 | -34 | 24 |
|  | SMG |  | R |  | 5.23 | 68 | -46 | 12 |
|  | AG | 39 | R |  | 3.68 | 62 | -46 | 22 |
|  | **SPL** | 7 | L | 789 | 5.57 | -18 | -56 | 66 |
|  | LOC | 7 | L |  | 4.26 | -26 | -60 | 66 |
|  | **IFG, pars op.** | 44 | R | 1862 | 5.43 | 42 | 16 | 28 |
|  | Precentral gyrus | 6 | R |  | 5.17 | 38 | -4 | 56 |
|  | MFG | 6 | R |  | 4.99 | 40 | 2 | 44 |
|  | **MFG** | 9 | L | 217 | 4.93 | -36 | 20 | 26 |
|  | IFG, pars op. | 44 | L |  | 3.77 | -46 | 18 | 20 |
|  | **SFG** |  | L | 538 | 4.87 | -20 | -10 | 64 |
|  | Precentral gyrus |  | L |  | 4.40 | -36 | -6 | 50 |
|  | SMA | 6 | L |  | 3.46 | -8 | -10 | 56 |
|  | **STG** | 38 | R | 448 | 4.79 | 52 | 4 | -16 |
|  | Planum polare | 22 | R |  | 4.18 | 50 | -6 | -10 |
|  | Temporal pole | 38 | R |  | 4.08 | 48 | 6 | -26 |
|  | **SMA** | 6 | R | 592 | 4.68 | 10 | 2 | 52 |
|  | SFG | 6 | R |  | 4.45 | 18 | -2 | 74 |
|  | **SPL** | 7 | R | 392 | 4.47 | 22 | -54 | 76 |
|  | LOC | 7 | R |  | 4.30 | 26 | -64 | 66 |
|  | Precuneus | 7 | R |  | 3.63 | 12 | -48 | 59 |

S8. fMRI Results: Effect of task in a pooled sample

As a sanity check, we assessed the effect of task, i.e., no IPS modulation using a pooled sample, in a whole brain analysis (Figure 8.1; Table S8.1).

**Table S8.1.** Results of the effect of task in a whole brain analysis using a pooled sample. All significant clusters are reported from a non-parametric, cluster mass extent approach considering p < .05. Coordinates are reported in MNI space. BA: Brodmann Area; H: Hemisphere; L: Left; R: Right

| Effect | Region | BA | H | Cluster Size | t-value | x | y | z |
| --- | --- | --- | --- | --- | --- | --- | --- | --- |
| Task | | | | | | | | |
|  | **LOC** | 37 | R | 8457 | 15.16 | 48 | -70 | 2 |
|  | Occipital pole | 18 | R |  | 11.36 | 30 | -92 | 10 |
|  | SMG | 22 | R |  | 8.57 | 45 | -40 | 9 |
|  | Temporal occipital fusiform cortex | 37 | R |  | 8.21 | 43 | -53 | -10 |
|  | AG | 39 | R |  | 7.06 | 49 | -47 | 19 |
|  | ITG | 37 | R |  | 6.77 | 46 | -36 | -16 |
|  | **LOC** | 19 | L | 5376 | 14.00 | -46 | -78 | 8 |
|  | Occipital pole | 18 | L |  | 10.93 | -28 | -92 | 6 |
|  | Temporal fusiform cortex | 37 | L |  | 7.98 | -38 | -45 | -15 |
|  | Temporal occipital fusiform cortex | 37 | L |  | 7.06 | -36 | -46 | -13 |
|  | SMG |  | L |  | 4.01 | -46 | -47 | 14 |
|  | **IFG, pars op.** | 44 | R | 2669 | 8.38 | 44 | 16 | 26 |
|  | Precentral gyrus | 44 | R |  | 8.00 | 40 | 10 | 28 |
|  | IFG, pars tri. | 9 | R |  | 7.82 | 54 | 26 | 20 |
|  | MFG | 44 | R |  | 6.58 | 52 | 18 | 32 |
|  | Frontal pole | 47 | R |  | 6.12 | 48 | 36 | -2 |
|  | **STG** | 22 | R | 409 | 6.29 | 50 | -8 | -10 |
|  | **IFG, pars tr.** |  | L | 394 | 5.27 | 17 | 76 | 49 |
|  | IFG, pars op. | 44 | L |  | 4.69 | -42 | 12 | 26 |
|  | MFG | 44 | L |  | 4.16 | -46 | 19 | 26 |

**Figure S8.1.** The clusters significantly modulated by task activation (blue) in a pooled sample from a whole brain analysis.


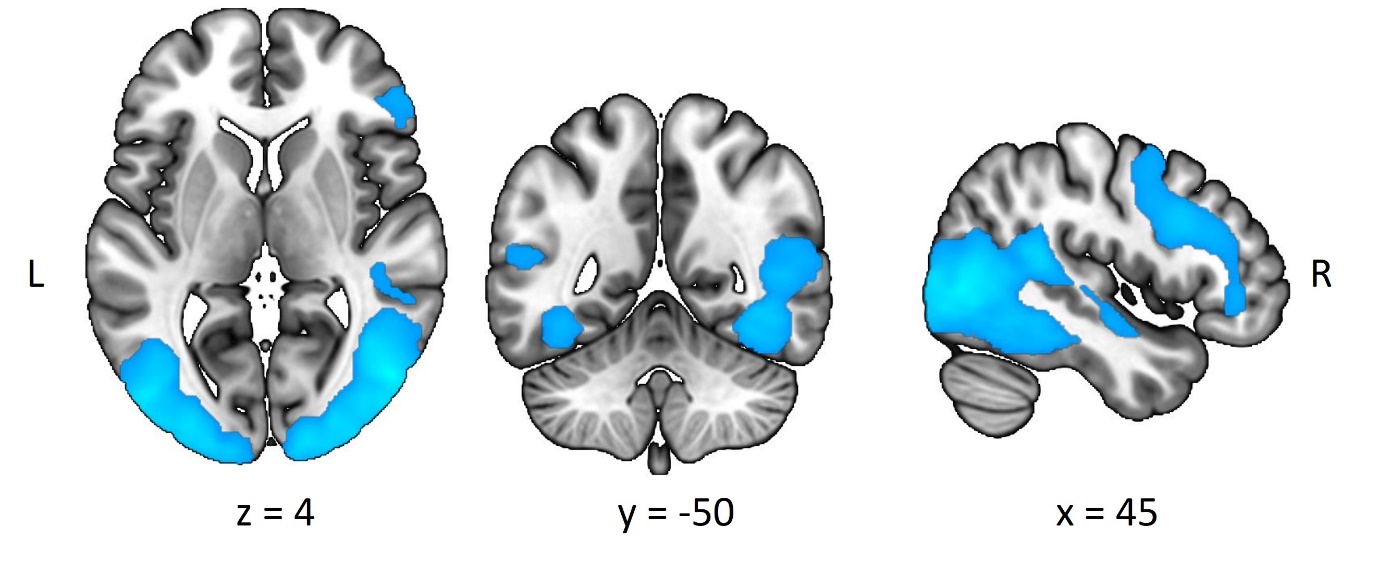

Supplement: Supplementary file 1 — Data S1. Supporting Information. [file HBM-45-e70052-s001.docx]
